# DNA methylation in ductal carcinoma in situ related with future development of invasive breast cancer

**DOI:** 10.1186/s13148-015-0094-0

**Published:** 2015-07-25

**Authors:** Kevin C. Johnson, Devin C. Koestler, Thomas Fleischer, Panpan Chen, Erik G. Jenson, Jonathan D. Marotti, Tracy Onega, Vessela N. Kristensen, Brock C. Christensen

**Affiliations:** Department of Pharmacology and Toxicology, Geisel School of Medicine at Dartmouth, HB 7650, Remsen 611, Hanover, NH 03755 USA; Department of Epidemiology, Geisel School of Medicine at Dartmouth, HB 7650, Remsen 611, Hanover, NH 03755 USA; Department of Biostatistics, University of Kansas Medical Center, Kansas City, KS USA; Department of Genetics, Institute for Cancer Research, Oslo University Hospital Radiumhospitalet, Oslo, Norway; The K.G. Jebsen Center for Breast Cancer, Institute for Clinical Medicine, Faculty of Medicine, University of Oslo, Oslo, Norway; Department of Pathology, Geisel School of Medicine at Dartmouth, Hanover, NH 03755 USA; Department of Data Science, Geisel School of Medicine at Dartmouth, Hanover, NH 03755 USA; The Dartmouth Institute, Geisel School of Medicine at Dartmouth, Lebanon, NH 03766 USA; Department of Clinical Molecular Biology (EpiGen), Medical Division, Akershus Hospital, Lørenskog, Norway; Department of Community and Family Medicine, Geisel School of Medicine at Dartmouth, Hanover, NH 03755 USA

**Keywords:** Breast, Methylation, Array, DCIS, Cancer, TCGA

## Abstract

**Background:**

Ductal carcinoma in situ (DCIS) is a heterogeneous, pre-invasive lesion associated with an increased risk for future invasive ductal carcinoma. However, accurate risk stratification for development of invasive disease and appropriate treatment decisions remain clinical challenges. DNA methylation alterations are early events in the progression of cancer and represent emerging molecular markers that may predict invasive recurrence more accurately than traditional measures of DCIS prognosis.

**Results:**

We measured DNA methylation using the Illumina HumanMethylation450K array of estrogen-receptor positive DCIS (*n* = 40) and adjacent-normal (*n* = 15) tissues from subjects in the New Hampshire Mammography Network longitudinal breast imaging registry. We identified locus-specific methylation differences between DCIS and matched adjacent-normal tissue (95,609 CpGs, *Q* < 0.05). Among 40 DCIS cases, 13 later developed invasive disease and we identified 641 CpG sites that exhibited differential DNA methylation (*P* < 0.01 and median |∆β| > 0.1) in these cases compared with age-matched subjects without invasive disease. The set of differentially methylated CpG loci associated with disease progression was enriched in homeobox-containing genes (*P* = 1.3E-09) and genes involved with limb morphogenesis (*P* = 1.0E-05). In an independent cohort, a subset of genes with progression-related differential methylation between DCIS and invasive breast cancer were confirmed. Further, the functional relevance of these genes’ regulation by methylation was demonstrated in early stage breast cancers from The Cancer Genome Atlas database.

**Conclusions:**

This work contributes to the understanding of epigenetic alterations that occur in DCIS and illustrates the potential of DNA methylation as markers of DCIS progression.

**Electronic supplementary material:**

The online version of this article (doi:10.1186/s13148-015-0094-0) contains supplementary material, which is available to authorized users.

## Background

Ductal carcinoma in situ (DCIS) is a non-invasive precursor lesion to invasive ductal carcinoma and is frequently diagnosed upon screening mammography. In 2015, more than 60,000 DCIS diagnoses are expected in the USA, accounting for over 25 % of new breast cancer cases [1]. Importantly, the risk of developing invasive breast cancer among women with DCIS is significantly increased compared to the general population [2, 3]. Standard treatment of DCIS focuses on preventing the development of invasive breast cancer with at least local surgical excision to remove the proliferative lesion, although more extensive treatment including mastectomy is sometimes performed [4, 5]. The risk of local recurrence in patients treated with excision alone has been reported to be as high as 25 % over 10 years [6]. However, a majority of patients will never develop invasive disease once treated, and so there exists a growing concern that a subset of DCIS patients may be receiving excessive treatment [7, 8]. Overtreatment can potentially subject the patient to unnecessary risk of treatment, without potential benefit, and burden the health care system [9]. Thus, identifying differential risks of developing invasive breast cancer among DCIS patients may improve patient outcomes and reduce healthcare costs.

The deregulation of epigenetic modifications, such as DNA methylation, is an early event in breast carcinogenesis [10, 11, 5, 12]. The addition of a methyl group to a cytosine residue followed by a guanine (CpG) is recognized to be an important regulator of gene expression and chromosomal stability [13]. Although DNA methylation deregulation occurs early in carcinogenesis, the patterns of epigenetic alterations associated with development of invasive disease remain unclear. DNA methylation alterations are potential markers for DCIS outcome prediction and may identify cellular changes that modulate tumor progression. Previous studies have highlighted that DNA methylation alterations are widespread in invasive breast carcinoma and result in cellular dysfunction [14–18]. However, few studies have investigated the epigenetic alterations that occur in earlier stages of disease, and of those that have, many used a candidate gene approach [5, 19, 20, 10, 21, 22]. Accordingly, the identification of DNA methylation alterations that can inform risk of subsequent invasive disease has the potential to both impact clinical treatment decisions and improve our understanding of cancer progression.

In this study, we identified subjects through the New Hampshire Mammography Network (NHMN) and investigated DNA methylation patterns in estrogen-receptor (ER) positive DCIS samples for their relation with time to diagnosis of invasive breast cancer. We have made use of a unique resource in the NHMN, which follows patients longitudinally, to examine DCIS of women who did and did not develop invasive disease over the same period of time. Here, we have used genome-wide DNA methylation arrays to comprehvensively identify DCIS-specific alterations in DNA methylation that differ in these two groups. We subsequently extended our findings to independent populations of DCIS and invasive ductal carcinomas to further explore disease-specific methylation deregulation and provide evidence for the potential impact of these alterations on gene expression [15]. Through these analyses, we have identified epigenetic changes in ER positive DCIS that may contribute to increased risk of developing invasive breast cancer.

## Results

### Unsupervised clustering of DNA methylation in ductal carcinoma in situ

**Fig. 1:**
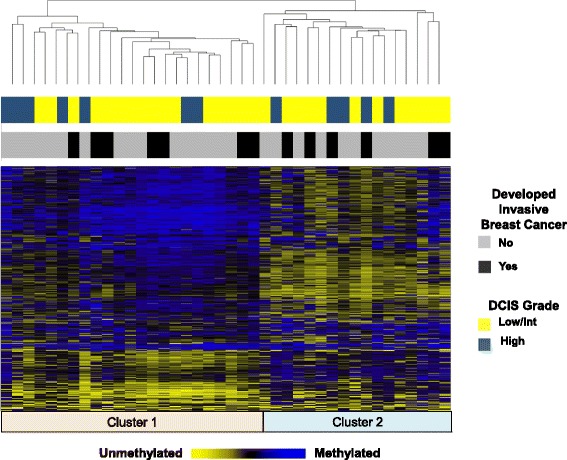
Unsupervised clustering and heat map of DNA methylation in DCIS samples (*n* = 40). Unsupervised hierarchical clustering heat map based on Manhattan distance and average linkage of the 10,000 CpG loci with the greatest variance

Patient demographic data and DCIS pathologic characteristics are presented in Table 1. To characterize DNA methylation patterns of DCIS tissues (*n* = 40) and available matched adjacent-normal samples (*n* = 15), we measured genome-wide DNA methylation using the Infinium HumanMethylation450 BeadArray. The scheme of our analysis strategy for identifying deregulated methylation in DCIS is depicted in Additional file 1: Figure S1. First, we performed unsupervised hierarchical clustering of the 10,000 most variable DNA methylation loci and observed two distinct clusters of methylation profiles among DCIS samples (Figure. 1). The optimal number of two clusters was verified by a resampling based unsupervised consensus clustering method (Additional file 2: Figure S2) [23]. There was no significant enrichment for membership in either methylation cluster for DCIS grade, subsequent diagnosis of invasive breast cancer, or subject age. Next, we assessed locus-specific patterns of differential methylation between DCIS tissues and matched adjacent-normal breast tissue at single base resolution fitting linear mixed effects models across 55 tissues (40 DCIS and 15 adjacent normal). This analysis revealed profound alterations in global methylation patterns (Additional file 3: Figure S3) with the identification of 95,609 differentially methylated CpGs significant after correction for multiple comparisons (*Q* < 0.05, 20.3 % of CpGs).

**Table 1:** New Hampshire Mammography Network patient and DCIS characteristics

	All DCIS	No invasive BRCA	Invasive BRCA	
Covariate	*n* = 40	*n* = 27 (67.5 %)	*n* = 13 (32.5 %)	*P* value
Age (years)				
Range	37–78	37–78	38–76	
Median	59	63	52	
Mean (sd)	58.2 (12.8)	59.1 (13.0)	56.5 (12.9)	0.55
Follow-up time (years)				
Median	7.01	8.55	2.94	
Range	0.94–15.49	2.85–15.49	0.94–10.33	1.60E-04
Grade				
Low/Intermediate (%)	28 (70.0)	17 (63.0)	11 (84.6)	
High	12 (30.0)	10 (37.0)	2 (15.4)	0.27
Tumor size (cm)				
Median	1.20	1.4	1.15	
Range	0.15–7.5	0.2–7.5	0.15–2.1	
Missing	8	5	3	0.64
Family history of breast cancer				
No (%)	25 (62.5)	17 (63.0)	8 (61.5)	
Yes	15 (37.5)	10 (37.0)	5 (38.5)	1.0
Menopausal status				
Pre-menopausal (%)	15 (37.5)	9 (33.3)	6 (46.2)	
Post-menopausal	25 (62.5)	18 (66.7)	7 (53.8)	0.59

T-tests or Wilcoxon tests were applied for numeric variables, while Fisher’s exact test was used for count data

*P* value represents results from univariate tests between study groups

### Methylation patterns and development of invasive breast cancer

**Fig. 2:**
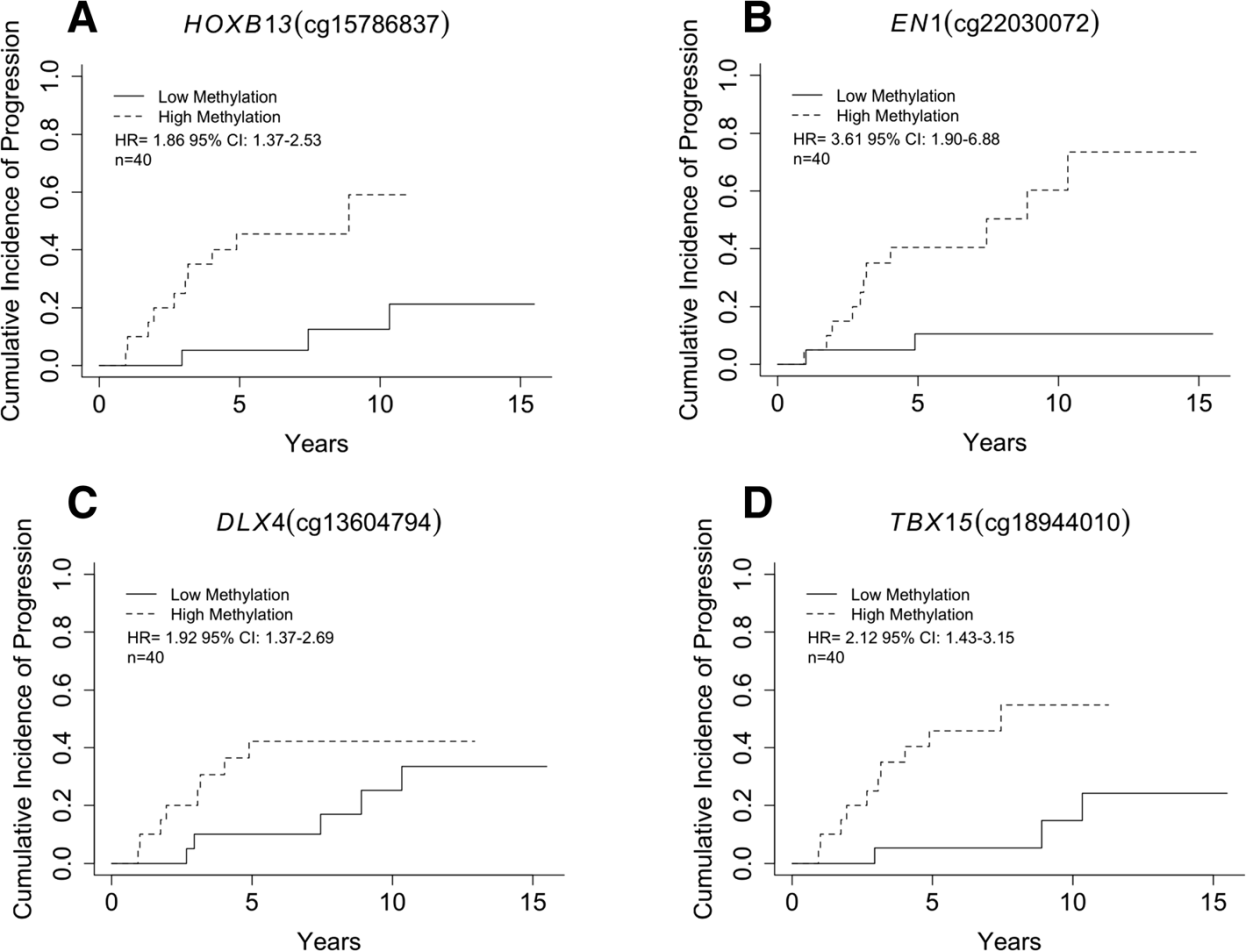
Cumulative incidence of invasive breast cancer diagnosis stratified by the median methylation in DCIS of each CpG into high or low methylation groups. **a** Increased methylation in DCIS of a CpG in the south shore of a *HOXB13* promoter CpG island is associated with invasive breast cancer outcome (HR = 1.86, 95 % CI, 1.37–2.53) (high-methylation beta-value range, 0.14–0.71**,** low-methylation beta-value range, 0.03 - 0.13). **b** Increased methylation in DCIS of a gene body CpG island site in *EN1* is associated with invasive breast cancer outcome (HR = 3.61, 95 % CI, 1.90–6.88) (high-methylation beta-value range, 0.27–0.58**,** low-methylation beta-value range, 0.069 - 0.27). **c** Increased methylation in DCIS of a gene body CpG island site in *DLX4* is associated with invasive breast cancer outcome (HR = 1.92, 95 % CI, 1.37–2.69) (high-methylation beta-value range, 0.31–0.78**,** low-methylation beta-value range, 0.078–0.31). **d** Increased methylation in DCIS of a CpG site in the south shore of a CpG island in the 5′’UTR of *TBX15* is associated with invasive breast cancer outcome (HR = 2.12, 95 % CI, 1.43–3.15) (high-methylation beta-value range, 0.42–0.82**,** low-methylation beta-value range, 0.13 - 0.39)

To identify DNA methylation related with subsequent development of invasive breast cancer, we fit Cox proportional hazards models to examine the association between time-to-event data and the DNA methylation values at each CpG locus independently (beta-value) and adjusted for age and DCIS grade. We identified 641 CpGs, representing 397 genes, whose methylation was associated with development of invasive disease (*P* < 0.01, with at least 10 % change in methylation, Additional file 4: Table S1). The strongest associations with development of invasive ductal carcinoma (IDC) are presented in Table 2 and representative plots of cumulative incidence for high and low methylation groups (stratified by median DNA methylation values at a given CpG) are shown in Fig. 2. Notably, among the 641 CpG progression-associated loci there were 276 CpGs (Additional file 4: Table S1) that exhibited differential methylation between DCIS and adjacent normal as well as further directionally consistent methylation alterations in subjects that ultimately progressed to invasive disease (Additional file 5: Figure S4 for representative plots). As expected, a stratified analysis determined that the strength of association was stronger with an ipsilateral IDC to DCIS outcome compared with a contralateral IDC outcome (Additional file 6: Table S2). Among DCIS patients with a subsequent diagnosis of invasive breast cancer, sidedness was not related with the methylation clusters defined in Fig. 1.

**Table 2:** CpGs that possess the strongest associations between methylation levels and development of invasive ductal carcinoma

Illumina cg ID	Chromosome	Genomic location	Gene^a^	Gene region	HR (95 % CI)^b^	*P* value
cg15786837	17	46806935	*HOXB13*	TSS1500	1.86 (1.37–2.53)	6.97E-05
cg22030072	2	119603673	*EN1*	Body	3.61 (1.90–6.88)	9.54E-05
cg13604794	17	48050338	*DLX4*	Body	1.92 (1.37–2.69)	1.47E-04
cg18944010	1	119522855	*TBX15*	5′UTR	2.12 (1.43–3.15)	1.88E-04
cg01002529	3	69591807			2.35 (1.50–3.70)	2.23E-04
cg08261841	7	142982463	*TMEM139*	5′UTR	0.56 (0.41–0.77)	2.82E-04
cg02926307	12	106520198	*NUAK1*	Body	0.40 (0.25–0.67)	3.60E-04
cg05075579	11	121421206	*SORL1*	Body	3.60 (1.77–7.30)	3.99E-04
cg15350661	12	322641	*SLC6A12*	5′UTR	2.31 (1.45–3.70)	4.10E-04
cg02770983	11	31822341	*PAX6*	Body	2.11 (1.39–3.19)	4.66E-04
cg00232535	7	5593287			0.47 (0.30–0.72)	4.79E-04
cg18343474	1	229566908			1.93 (1.33–2.80)	4.87E-04
cg00725138	22	19716556			0.50 (0.34–0.74)	5.34E-04
cg03695909	X	47346601			0.30 (0.15–0.59)	5.49E-04
cg26236177	14	61110649			1.77 (1.28–2.44)	5.60E-04
cg13434989	13	78493305	*EDNRB*	TSS1500	1.83 (1.30–2.58)	5.67E-04
cg24006505	6	41337238			1.95 (1.33–2.58)	6.04E-04
cg04636194	X	49594154	*PAGE4*	5′UTR	0.43 (0.27–0.70)	6.07E-04
cg23429749	14	102050815			0.47 (0.31–0.73)	6.12E-04
cg05231018	17	46806763	*HOXB13*	TSS1500	1.70 (1.25–2.29)	6.18E-04
cg02492708	16	2005062	*RPL3L*	TSS1500	0.50 (0.34–0.75)	6.24E-04
cg21211271	1	6265071	*RNF207*	TSS1500	2.37 (1.44–3.90)	6.48E-04
cg16009714	17	70241415			3.48 (1.70–7.12)	6.60E-04
cg00321709	10	135341933	*CYP2E1*	Body	2.04 (1.35–3.08)	6.69E-04
cg20401252	2	5834062	*SOX11*	1stExon	1.90 (1.31–2.73)	6.70E-04
cg19827511	14	93580644	*ITPK1*	Body	0.56 (0.39–0.78)	6.86E-04
cg10827488	11	113953838	*ZBTB16*	Body	3.42 (1.68–6.96)	6.97E-04
cg04327247	9	100564659			0.50 (0.34–0.75)	7.84E-04
cg26511386	7	142982262	*TMEM139*	5′UTR	0.52 (0.36–0.76)	8.02E-04
cg00332067	12	55004429	*GLYCAM1*	TSS200	2.10 (1.36–3.24)	8.30E-04

^a^Missingness represents a CpG locus that does not track to a gene

^b^For each 10 % change in methylation, Cox proportional hazards models were adjusted for subject age and DCIS grade

We next sought to identify common biological processes, pathways, and molecular functions with enrichment among genes with progression-related methylation using the set of 641 CpG loci. Among the 375 Database for Annotation, Visualization, and Integrated Discovery (DAVID) genes that had at least one of the 641 differentially methylated CpGs, an overrepresentation of homeobox genes (8.54-fold enrichment, *P* = 1.3E-09) and limb morphogenesis genes (4.9-fold enrichment, *P* = 1.0E-05) were identified (Additional file 7: Table S3). A majority (70.3 %, Additional file 4: Table S1) of progression-related CpGs are located within or in close proximity to genes. Nearly 50 % of progression-related CpGs that exhibited losses in methylation were open sea probes, while only 9.5 % (Additional file 8: Table S4) tracked to CpG Islands. In contrast, 31.4 % of CpG loci with gains in DNA methylation tracked to CpG Islands (Additional file 8: Table S4). To further examine the nature of progression-related methylation in DCIS, we evaluated the potential enrichment of genomic features among the 641 CpG loci. We observed a substantial enrichment of polycomb group target genes (PCGTs) (*P* = 7.3E-07, Table 3), open sea probes (*P* = 1.5E-04, Table 3), and informatically predicted enhancer regions (*P* = 1.8E-22, Table 3) among progression-related CpG loci. CpG loci that were located in CpG islands were depleted among progression-related loci (*P* = 9.8E-07, Table 3), and we did not observe enrichment of progression-related CpGs to CpG island shores (*P* = 0.06, Table 3), CpG island shelves (*P* = 0.36, Table 3), or transcription factor binding sites (TFBSs) (*P* = 0.89, Table 3).

**Table 3:** Enrichment of genomic features among 641 progression-related loci

A			
Polycomb Group Target Genes (PCGT)	PCGT Target	NonPCGT Target	
All array CpGs	49,054 (12.3 %)	348,546 (87.7 %)	
Progression-related CpGs	123 (19.2 %)	518 (80.8 %)	*P value = 7.3E-07
B			
Enhancer Regions	Enhancer	Nonenhancer	
All array CpGs	72,289 (18.2 %)	325,311 (81.8 %)	
Progression-related CpGs	176 (33.5 %)	465 (66.5 %)	*P value = 1.8E-22
C			
CpG Island Status	CpG Island	NonCpG Island	
All array CpGs	145,618 (36.6 %)	251,982 (63.4 %)	
Progression-related CpGs	176 (33.5 %)	465 (66.5 %)	*P value = 9.8E-07
D			
Open Sea	Open Sea	Nonopen Sea	
All array CpGs	125,650 (31.6 %)	271,950 (68.4 %)	
Progression-related CpGs	248 (38.7 %)	393 (61.3 %)	*P value = 1.5E-04
E			
CpG Island Shore	CpG Island Shore	NonCpG Island Shore	
All array CpGs	92,703 (23.3 %)	363971 (76.7 %)	
Progression-related CpGs	170 (26.5 %)	471 (73.5 %)	*P value = 6.1E-02
F			
CpG Island Shelf	CpG Island Shelf	NonCpG Island Shelf	
All array CpGs	33,629 (8.5 %)	363,971 (91.5 %)	
Progression-related CpGs	176 (7.3 %)	594 (92.7 %)	*P value = 0.35
G			
TFBSany	TFBS	NonTFBS	
All array CpGs	127 688 (32.2 %)	269 912 (67.8 %)	
Progression-related CpGs	204 (31.8 %)	437 (68.2 %)	*P value = 0.89

CpGs among the 641 progression-related loci in DCIS are enriched for localization to A Polycomb group target genes (PCGTs), B informatically predicted enhancer regions, C depleted for localization to CpG Islands, and D enriched at Open Sea probes. Progression-related loci did not exhibit enrichment of E CpG Island shores, F CpG Island shores, or G any transcription factor binding site (TFBS)

*Fisher’s exact test

### Extension to an independent set of DCIS and IDC samples

An independent cohort of unmatched ER positive pure DCIS (*n* = 17) and ER positive invasive ductal carcinomas (IDC, *n* = 115) with methylation data from the same array platform was identified to validate differential methylation of progression-related genomic regions between DCIS and IDC. While data on subsequent diagnosis of invasive breast cancer were not available for DCIS cases in the independent cohort, increasing invasive potential may be reflected in the methylation changes that exist between DCIS and IDC. Comparing DCIS and IDC in the independent cohort revealed 3000 CpGs that were differentially methylated (*P* < 0.01, at least 10 % change, Additional file 9: Table S5). Overall, four CpGs were significantly differentially methylated in both populations (*P* < 0.01, at least 10 % change, Additional file 10: Table S6). In an expanded gene-level analysis, there were 397 genes related with the 641 CpG progression-associated loci. Among this gene list were 72 genes (18.1 %, Additional file 9: Table S5) that experienced differential methylation between DCIS and IDC in the independent cohort. An unsupervised clustering of DNA methylation from the 72 shared genes (77 CpGs) distinguished a subset of DCIS subjects who later developed invasive disease (Additional file 11: Figure S5). In the independent cohort results, 28 out of 72 genes had a methylation change in the same genomic region (i.e., promoter region, gene body region, and same direction as CpGs that were identified in the discovery cohort. Restriction to these 28 genes did not improve clustering (Additional file 11: Figure S5). A DAVID analysis revealed that genes associated with mesenchymal cell differentiation experienced the greatest enrichment among the 72 gene-set, but the finding did not reach statistical significance (1.73-fold enrichment, *P* = 0.72).

### Assessment of methylation alterations and gene expression in a set of primary TCGA breast tumors

**Fig. 3:**
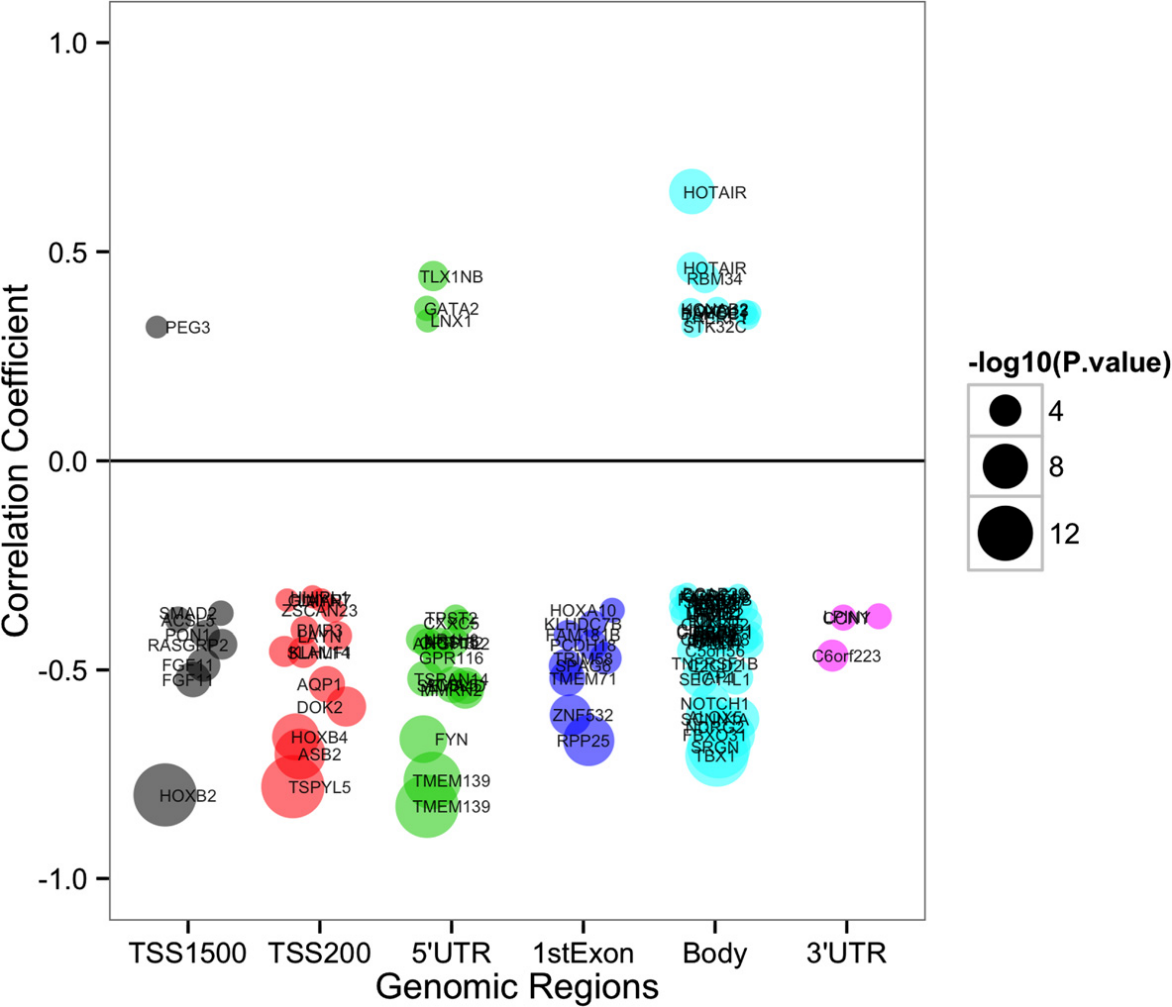
Genes whose expression levels correlated with methylation level of progression-related CpGs from Stage I ER positive TCGA breast tumors. Bubble points represent genes that demonstrate a significant relationship between methylation of a CpG and expression of its nearby gene in a genomic-context dependent manner. Increasing bubble diameter corresponds with decreasing *P* value. CpG position relative to gene location is plotted versus correlation coefficient between CpG methylation and gene expression

Next, we investigated the relation of methylation alteration with gene expression for progression-related CpGs. Methylation of progression-related CpGs was strongly correlated with gene expression in Stage I invasive breast cancers; 101 out of 384 CpGs were significantly correlated (*P* < 0.01, Additional file 12: Table S7). Among the strongest associations between methylation and expression were CpG sites associated with *TMEM139*, *HOXB2*, and *TBX1* genes as well as the long non-coding RNA *HOTAIR* (Fig. 3). As expected, a large majority (87.1 %) of significant associations between methylation and gene expression were negative correlations. The dependency of regulation of gene expression by methylation on genomic context was also apparent. A majority of the CpGs whose methylation was positively correlated with expression were located in gene bodies (69.3 %), while those CpGs negatively correlated with expression were equally likely to be associated with promoter regions and first exons of genes (48.9 %) or the gene body (47.7 %).

## Discussion

A challenge in the management of DCIS is variability in risk of developing invasive breast cancer. Decisions regarding treatment are complicated by a lack of reproducible clinical and pathologic factors that can reliably predict risk of future invasive disease after surgical excision. Identification of molecular alterations differentiating lesions that will remain indolent from those that will likely acquire an invasive phenotype begins to address a critical need to provide more accurate risk assessment. Emerging evidence from the initial stages of cancer development suggests that the patterns of DNA methylation, a stable mark capable of transcriptional control, are deregulated early and may serve as a predictor of malignant potential [19, 21, 5]. The present study aimed to characterize the patterns of epigenetic deregulation that occur in the early stages of breast cancer by investigating genome-wide methylation patterns in DCIS and adjacent-normal tissue. We identified methylation of CpG loci that were strongly associated with development of invasive breast cancer. Notably, some of these alterations exhibited further change when comparing DCIS with invasive tumors. The CpG loci related to disease progression were highly enriched for homeobox family of proteins and genomic features such as polycomb group gene targets. Importantly, CpGs with progression-related changes in methylation in DCIS had strong correlations with gene expression in an independent set of early stage ER positive breast cancers, suggesting that differential methylation of these sites may contribute to an increased risk of invasion through aberrations in gene expression.

DCIS represents a direct precursor to invasive ductal carcinoma. Altered patterns of DNA methylation have the ability to the ability to modify the regulation of gene expression, impact chromosomal instability, and have frequently been observed in the early stages of breast cancer [18]. However, the precise timing of molecular alterations during tumorigenesis and how these changes to the breast epigenome may influence the risk of becoming invasive cancer remain incompletely defined. In prior work and in this study, extensive epigenetic differences were observed between adjacent-normal and DCIS tissues, confirming that pre-invasive DCIS lesions harbor a high degree of disruption in the methylation patterning of ductal epithelial cells [24]. The striking difference between DCIS and tissues with no histologic evidence of malignancy provides additional evidence that epigenetic alterations in breast cancer are early events. Importantly, the delineation of methylation changes in DCIS compared to normal tissue helps to differentiate those early events that prime cells with oncogenic insults from later events (i.e., events measured in invasive breast cancer) that may increase malignant potential.

It is clear that pronounced molecular alterations occur during the normal to DCIS transition; however, genetic alterations to explain the divergent gene expression patterns between in situ and invasive breast carcinomas have not been observed [25]. Epigenomic changes represent potential molecular attributes that define whether a carcinoma remains indolent or shifts toward an invasive phenotype. Indeed, we observed that altered DNA methylation associated with an invasive disease diagnosis preferentially targeted gene groups were involved with key developmental pathways. For instance, DNA methylation of informatically predicted enhancer regions was enriched among progression-related CpGs. Interestingly, dysregulation of DNA methylation at these distal regulatory sites has previously been related with the expression of cancer genes [26]. Homeobox genes and other developmental transcription factors become preferential targets of de novo methylation in DCIS, consistent with previous associations between homeobox gene methylation and recurrence in invasive breast cancers [21, 27]. Together, these data provide evidence that perturbations in methylation among homeobox genes may promote a transformed and invasive phenotype. Similarly, biological processes that were not shown to be enriched via DAVID, but present among the differentially methylated loci, included Polycomb group protein target genes (PCGTs). Polycomb group proteins reversibly repress their target genes that are required for differentiation and necessary to promote the self-renewal potential of stem cells [28]. In our study, the PCGTs were substantially more likely to have progression-specific DNA hypermethylation than non-targets providing evidence that acquisition of DNA methylation at these genes could predispose cells to malignant transformation. In summary, genes involved in developmental processes are dysregulated early in disease development and may represent critical events that are exacerbated as ductal cells progress to a more malignant phenotype.

At the gene level, the strongest locus-specific associations with progression tracked to genes with previously demonstrated involvement in carcinogenesis. For example, overexpression of *EN1* is associated with pro-survival in basal-like breast cancer [29]; *HOXB13* hypermethylation has been shown to be a late event in breast tumorigenesis and is associated with invasiveness [30, 31]*;* increased expression of *DLX4* reduces invasion in vitro and metastasis in vivo [32]*;* and increased *TBX15* methylation was a poor prognostic indicator in prostate cancer [33]. Moreover, there were 276 CpG loci including those that track to the *HOXB13*, *DLX4,* and *TBX15* genes that exhibited a significant gain or loss of methylation from normal to DCIS and were further hypermethylated or hypomethylated in lesions from women with a subsequent diagnosis of invasive disease. Additional deregulation of methylation at these sites suggests that a greater proportion of cells harbor alterations associated with an invasive phenotype. Based on these preliminary results, it seems reasonable to envision a clinical scenario where leveraging both pathologic and molecular characteristics of DCIS and surrounding normal tissues will contribute to decisions of whether more or less aggressive treatment and/or monitoring is warranted.

The results from comparing methylation in an independent set of DCIS tissues with IDC indicated that differences between invasive disease and DCIS overlap with those that potentiate development of an invasive phenotype. Our DAVID analysis—despite being best suited for gene sets between 100–2000 genes—of the 72 genes shared between data sets suggests that epigenetic deregulation of genes involved in cellular development and differentiation is likely a defining feature progression to invasive disease [34]. Further, alterations that are critical for development of an invasive phenotype may occur in DCIS and once the cancerous cells escape from the duct additional epigenetic programming may be required for further progression or metastasis. Regardless, in early stage invasive breast tumors from TCGA, we observed that progression-related CpGs exhibit strong correlations with gene expression. Our results provide evidence that the acquisition or loss of DNA methylation at the identified progression-related CpGs has a functional impact on gene expression. Examples of potential critical methylation changes include the following: *GATA2* expression (methylation was positively correlated with expression among the TCGA samples) has been shown to be elevated in breast cancer and serves to repress *PTEN* expression [35], *HOXB2* for which increased methylation is associated with bladder cancer invasiveness [36], and the long non-coding RNA *HOTAIR,* which demonstrated increases in methylation associated with progression and was also positively correlated with expression in TCGA tumors*.* Interestingly, *HOTAIR* is a long non-coding RNA responsible for Hox gene silencing. *HOTAIR* is increased in expression in primary breast tumors and metastases, and loss of *HOTAIR* can inhibit cancer invasiveness [37]. Early methylation events that target homeobox genes in DCIS may, in part, be responsible for the aberrant gene expression of homeobox genes that has been observed in cancer [38]. Overall, we observed that progression-related methylation patterning is strongly related with transcription of genes that are known to be associated with the invasive phenotype.

The major objective of our work was to determine epigenetic events that contribute to development of invasive breast cancer. Our study is strengthened by the use of time-to-event data for subsequent diagnosis of invasive breast cancer. The longitudinal aspect of the cohort is an important distinction from previous cross-sectional approaches that have examined DCIS and invasive disease with a lack of clinical follow-up. In spite of this, each cohort in our investigation had a limited sample size. Therefore, future studies of methylation alterations in additional DCIS specimens with follow-up data are needed to further confirm and more precisely define potential biomarkers of risk stratification among DCIS subjects. In addition, future population-based studies that collect samples from the same individual across the spectrum of disease and investigate the relation of DNA methylation with breast cancer risk factors may also be informative for enhanced prediction of risk for disease progression.

## Conclusions

Our approach identified methylation alterations present in DCIS that may contribute to breast tumorigenesis and development of invasive disease. Progression from DCIS to invasive disease represents a substantial increase in the risk of breast cancer mortality, and identification of disease progression markers for DCIS is needed. We have provided evidence for methylation alterations that are associated with development of invasive disease and defined specific epigenetic programs in DCIS disease progression that are particularly susceptible to deregulation. Further examination of these alterations is warranted to demonstrate their potential utility in defining risk for subsequent invasive disease and thus impact treatment decision-making.

### Availability of supporting data

Data for all NHMN DCIS DNA methylation microarray experiments are available on the National Center for Biotechnology Information’s gene expression omnibus under the accession number GSE66313.

## Methods

### Study design and patient population

Pure ductal carcinoma in situ (DCIS) samples for 43 subjects were identified through the New Hampshire Mammography Network (NHMN), under the approval of the Institutional Review Board. The NHMN is a state-based mammography registry that records information from breast imaging exams and subsequent pathology, cancer, and vital status outcomes for consenting women [39]. For the present report, the analyses were based on samples collected from women who underwent resection of a breast lesion at Dartmouth-Hitchcock Medical Center (Lebanon, NH, USA). Three DCIS samples were removed from analysis in the quality assessment and control steps of methylation data processing detailed below. Among ER positive DCIS cases (*n* = 40), there were 13 patients with a subsequent diagnosis of invasive ductal carcinoma (IDC) and 27 age-matched subjects with similar follow up time who did not have a subsequent diagnosis of IDC. The time to progression and date of last known follow-up for those without subsequent diagnosis were recorded; median clinical follow-up for this cohort was 7 years. Slides from all subjects underwent central pathology review by a breast pathologist (JDM) to confirm diagnosis of ductal carcinoma in-situ and record histopathologic features. Multiple (at least two) 2-mm tissue cores were taken from the archived formalin-fixed paraffin-embedded (FFPE) blocks from selected areas of DCIS and adjacent-normal tissue for DNA extraction and bisulfite modification. The available block with the greatest, homogeneous amount of target tissue was selected for DNA extraction. The patient-matched normal breast tissues adjacent to DCIS did not have any histologic evidence of malignancy and were randomly selected from 15 subjects. Patients were not treated with neoadjuvant therapy, and subsequent invasive ductal carcinoma samples from matched DCIS subjects were not evaluated in this study. Material for the independent population was obtained from ER positive pure DCIS (*n* = 17) and ER positive IDC (*n* = 115) as described in GSE60185 [24]. All samples were taken in compliance with the Helsinki Declaration.

### Array-based DNA methylation assessment and quality control

Formalin-fixed paraffin embedded tissue samples were disrupted before subsequent DNA purification using the TissueLyserII (Qiagen, Valencia, CA, USA) for 1 min at 30 Hz. DNA was then isolated using the QIAamp DNA formalin-fixed paraffin embedded tissue kit (Qiagen, Valencia, CA, USA) according to the manufacturer’s protocol. Genomic DNA was bisulfite modified using the EZ DNA methylation kit (Zymo Research, Irvine, CA) and bisulfite-converted DNA from formalin-fixed paraffin embedded (FFPE) samples were processed as described in the Infinium FFPE Restoration guide (Illumina, San Diego, CA, USA) [40–42]. A recent study established the minimal effect that FFPE restoration has on methylation values via the demonstration of a very strong correlation between methylation β-values from paired fresh frozen and FFPE tissues [42]. After FFPE restoration, the resulting material was used as input for the hybridization on the Infinium HumanMethylation450 BeadChip (Illumina, San Diego, CA, USA), which has demonstrated high concordance with other bisulfite modification techniques such as pyrosequencing [17, 43–45]. Samples were randomized to plates and subjected to epigenome-wide DNA methylation assessment. The methylation status for each CpG locus was calculated as the ratio of fluorescent signals (*β* = Max (M, 0) / [Max(M,0) + Max(U,0) + 100]), ranging from 0 (non-methylated) to 1 (completely methylated), using average probe intensity for the methylated (M) and unmethylated (U) alleles. For preprocessing of the methylation data, we used the Chip Analysis Methylation Pipeline (ChAMP version 0.98.3) package in R [46]. Briefly, ChAMP takes the raw IDAT files from the arrays using data import, assesses the quality of probes and samples, provides adjustment for probe type bias, and allows for adjustment for batch-effects. As a part of the quality control step, three DCIS samples were removed because greater than 20 % of probes among these samples had a detection *P*-value greater than 0.01. In total, there were 40 DCIS and 15 adjacent-normal samples that passed quality control (QC) and were included in subsequent analyses. Probes were dropped from future analyses if the median detection *P*-value was >0.01 across all samples, which left a total of 397,600 out of 485,577 probes that passed QC. In the ChAMP package, we implemented beta-mixture quantile normalization (BMIQ) to adjust the data for bias introduced by the Infinium type 2 probe design [47]. Next, we corrected for potential batch-effects by applying the ComBat normalization method using the R-package “sva” [48]. Data for all DNA methylation microarray experiments are available on National Center for Biotechnology Information’s gene expression omnibus [35] in accordance with MIAME under the accession number GSE66313.

### Statistical analysis

#### Data assembly

All methylation data were analyzed using the R software environment, version 3.0.3 (www.r-project.org).

#### Unsupervised hierarchical clustering

Hierarchal clustering was based on Manhattan distance and average linkage of the 10,000 most variable CpG loci. The optimal number of clusters was established based on 1000 resampling interactions of K-means clustering for *K* = 2,3,4,5, with Euclidean distance being the distance metric implemented in the “ConsensusClusterPlus” R package [49].

#### Locus-by-locus analysis for detecting differentially methylated CpG loci

Locus-specific patterns of differential methylation between DCIS tissues (*n* = 40) and matched adjacent-normal breast tissue (*n* = 15) were identified via linear mixed effects models fit to each CpG independently, modeling logit-transformed *β*-values as the dependent variable and tissue-type and subject age as the independent variables. The linear mixed effects models included a random effect term for subject to account for repeat measures on the same subject. *P* values obtained from our linear mixed effects models were adjusted for multiple testing using false discovery rate estimation. The *Q* values were computed by the “qvalue” package in R and a *Q* value < 0.05 was deemed statistically significant. To identify epigenetic alterations associated with development of invasive disease among DCIS patients, locus-specific differences in DNA methylation of DCIS samples were examined using Cox proportional hazards models adjusted for subject age and DCIS grade (low/intermediate or high) [50]. To reduce Type I error and maintain sensitivity of discovery, we selected CpGs whose methylation was associated with development of invasive breast cancer at *P* < 0.01 and then limited our analyses to those CpG sites that exhibited a median |Δβ| greater than 0.1. We also implemented a stratified Cox proportional hazards analysis to explore whether there were different strengths in association between cases of ipsilateral (*n* = 7) and contralateral (*n* = 6) to DCIS.

#### Independent cohort of pure DCIS and pure IDC

Preprocessing and normalization of this data have previously been described above (ChAMP), and the raw data is available in Gene Expression Omnibus with accession number GSE60185. To identify epigenetic alterations that differentiate disease states (i.e., IDC compared with DCIS), we fit via linear models to each CpG independently, modeling logit-transformed *β*-values as the dependent variable and disease state as the independent variable.

#### The Cancer Genome Atlas data

Level 3 normalized Illumina Infinium Human Methylation450 BeadChip and RNASeqV2 rsem.genes.normalized_results data were downloaded from the TCGA (http://cancergenome.nih.gov/). All Stage I ER positive breast cancers from Caucasians were selected (*n* = 65). Expression data and CpG sites from the 641 differentially methylated loci were paired by gene symbol for the 65 samples, resulting in a total of 384 unique methylation and expression pairs used in the correlation analysis. The relation of methylation with gene expression was evaluated with Spearman correlations.

#### Enrichment analyses of biological pathways and common sequence features

The Database for Annotation, Visualization, and Integrated Discovery was used for an analysis of molecular pathways [34, 51]. In the DAVID analysis, the set of genes represented on the Illumina HumanMethylation450 array that remained after QC was used as the referent set and the set of genes associated with 641 progression-related CpG loci composed the gene set tested. Enrichment analyses of common sequence features among progression-related CpG loci were performed using two-tailed Fisher’s exact tests. The Polycomb group target genes (PCGT) status for a given CpG loci was based on whether the gene associated with that CpG was described as a PcG target in previously published works [52–55]. Putative transcription factor binding sites (TFBSs) located within 50 bp of progression-associated methylation of loci were obtained from the *tfbsConsSites* track of the UCSC Genome browser [56].

## Additional files

Additional file 1:
**Supplemental Figure S1**. Diagram of analytic strategy used to identify deregulated epigenetic patterns in DCIS and their relation with invasive breast cancer development. Additional file 2:
**Supplemental Figure S2**. Consensus clustering of 40 samples and 10,000 most variable CpGs identified 2 clusters as the optimal number. Additional file 3:
**Supplemental Figure S3**. Results from the locus-by-locus examination of differential methylation between DCIS and normal-adjacent to DCIS tissues. Additional file 4:
**Supplemental Table S1**. 641 CpG sites significantly associated with development of invasive ductal carcinoma. Additional file 5:
**Supplemental Figure S4**. A total of 276 CpGs among the 641 progression-related loci experienced additional DNA methylation deregulation in the same direction that differentiated DCIS from normal-adjacent tissue. Additional file 6:
**Supplemental Table S2**. CpGs that met the significance thresholds among the 641 progression-related loci when stratifying patients that developed IDC ipsilateral or contralateral to DCIS. Additional file 7:
**Supplemental Table S3**. Enriched annotation terms among differentially methylated genes. Additional file 8:
**Supplemental Table S4**. Distribution of progression-related CpGs across CpG Island status stratified by gains or losses of DNA methylation in DCIS subjects with an eventual diagnosis of IDC. Additional file 9:
**Supplemental Table S5**. CpGs that experienced differential methylation between DCIS (n=17) and IDC (n=115) in the Norwegian cohort and gene overlap for the 72 genes whose methylation was associated with development of invasive disease in NHMN. Additional file 10:
**Supplemental Table S6**. Overlap of 4 CpGs that exhibit differential methylation in both the DCIS progression and ER+ DCIS/IDC populations. Additional file 11:
**Supplemental Figure S5**. Unsupervised clustering heat map based on Manhattan distance and average linkage of CpGs that track to the 72 genes and 28 genes. Additional file 12:
**Supplemental Table S7**. Correlations of methylation with gene expression from TCGA stage I ER+ breast tumors (n=65).

## Abbreviations

CpG: Cytosine guanine dinucleotide
DCIS: ductal carcinoma *in situ*
FFPE: formalin-fixed paraffin-embedded
ER: estrogen receptor
TCGA: The Cancer Genome Atlas
NHMN: New Hampshire Mammography Network
QC: quality control
PCGT: polycomb group protein target

## References

[1] Siegel RL, Miller KD, Jemal A (2015). Cancer statistics, 2015. CA Cancer J Clin.

[2] Warren JL, Weaver DL, Bocklage T, Key CR, Platz CE, Cronin KA (2005). The frequency of ipsilateral second tumors after breast-conserving surgery for DCIS: a population based analysis. Cancer.

[3] Hartmann LC, Sellers TA, Frost MH, Lingle WL, Degnim AC, Ghosh K (2005). Benign breast disease and the risk of breast cancer. N Engl J Med.

[4] Duggal S, Julian TB (2013). A multigene expression assay to predict local recurrence risk for ductal carcinoma in situ. J Natl Cancer Inst.

[5] van Hoesel AQ, Sato Y, Elashoff DA, Turner RR, Giuliano AE, Shamonki JM (2013). Assessment of DNA methylation status in early stages of breast cancer development. Br J Cancer.

[6] Group EBCC, Group ER, Bijker N, Meijnen P, Peterse JL, Bogaerts J (2006). Breast-conserving treatment with or without radiotherapy in ductal carcinoma-in-situ: ten-year results of European Organisation for Research and Treatment of Cancer randomized phase III trial 10853—a study by the EORTC Breast Cancer Cooperative Group and EORTC Radiotherapy Group. J Clin Oncol Off J Am Soc Clini Oncol.

[7] Wickerham DL, Julian TB (2013). Ductal carcinoma in situ: a rose by any other name. J Natl Cancer Inst.

[8] Marshall E (2014). Breast cancer. Dare to do less Science.

[9] Pace LE, Keating NL (2014). A systematic assessment of benefits and risks to guide breast cancer screening decisions. JAMA.

[10] Muggerud AA, Ronneberg JA, Warnberg F, Botling J, Busato F, Jovanovic J (2010). Frequent aberrant DNA methylation of ABCB1, FOXC1, PPP2R2B and PTEN in ductal carcinoma in situ and early invasive breast cancer. Breast cancer research : BCR.

[11] Stefansson OA, Esteller M (2013). Epigenetic modifications in breast cancer and their role in personalized medicine. Am J Pathol.

[12] Veeck J, Esteller M (2010). Breast cancer epigenetics: from DNA methylation to microRNAs. J Mammary Gland Biol Neoplasia.

[13] Jones PA (2012). Functions of DNA methylation: islands, start sites, gene bodies and beyond. Nat Rev Genet.

[14] Kristensen VN, Vaske CJ, Ursini-Siegel J, Van Loo P, Nordgard SH, Sachidanandam R (2012). Integrated molecular profiles of invasive breast tumors and ductal carcinoma in situ (DCIS) reveal differential vascular and interleukin signaling. Proc Natl Acad Sci U S A.

[15] Cancer Genome Atlas N (2012). Comprehensive molecular portraits of human breast tumours. Nature.

[16] Fang F, Turcan S, Rimner A, Kaufman A, Giri D, Morris LG (2011). Breast cancer methylomes establish an epigenomic foundation for metastasis. Science translational medicine.

[17] Christensen BC, Kelsey KT, Zheng S, Houseman EA, Marsit CJ, Wrensch MR (2010). Breast cancer DNA methylation profiles are associated with tumor size and alcohol and folate intake. PLoS Genet.

[18] Widschwendter M, Jones PA (2002). DNA methylation and breast carcinogenesis. Oncogene.

[19] Klajic J, Fleischer T, Dejeux E, Edvardsen H, Warnberg F, Bukholm I (2013). Quantitative DNA methylation analyses reveal stage dependent DNA methylation and association to clinico-pathological factors in breast tumors. BMC Cancer.

[20] Moelans CB, Verschuur-Maes AH, van Diest PJ (2011). Frequent promoter hypermethylation of BRCA2, CDH13, MSH6, PAX5, PAX6 and WT1 in ductal carcinoma in situ and invasive breast cancer. J Pathol.

[21] Tommasi S, Karm DL, Wu X, Yen Y, Pfeifer GP (2009). Methylation of homeobox genes is a frequent and early epigenetic event in breast cancer. Breast cancer research : BCR.

[22] Pang JM, Dobrovic A, Fox SB (2013). DNA methylation in ductal carcinoma in situ of the breast. Breast cancer research: BCR.

[23] Monti S, Tamayo P, Mesirov J, Golub T (2003). Consensus clustering: a resampling-based method for class discovery and visualization of gene expression microarray data. Mach Learn.

[24] Fleischer T, Frigessi A, Johnson KC, Edvardsen H, Touleimat N, Klajic J (2014). Genome-wide DNA methylation profiles in progression to in situ and invasive carcinoma of the breast with impact on gene transcription and prognosis. Genome Biol.

[25] Polyak K (2010). Molecular markers for the diagnosis and management of ductal carcinoma in situ. J Natl Cancer Inst Monogr.

[26] Aran D, Sabato S, Hellman A (2013). DNA methylation of distal regulatory sites characterizes dysregulation of cancer genes. Genome Biol.

[27] Fackler MJ, Umbricht CB, Williams D, Argani P, Cruz LA, Merino VF (2011). Genome-wide methylation analysis identifies genes specific to breast cancer hormone receptor status and risk of recurrence. Cancer Res.

[28] Baylin SB, Ohm JE (2006). Epigenetic gene silencing in cancer—a mechanism for early oncogenic pathway addiction?. Nat Rev Cancer.

[29] Beltran AS, Graves LM, Blancafort P (2013). Novel role of Engrailed 1 as a prosurvival transcription factor in basal-like breast cancer and engineering of interference peptides block its oncogenic function. Oncogene.

[30] Shah N, Jin K, Cruz LA, Park S, Sadik H, Cho S (2013). HOXB13 mediates tamoxifen resistance and invasiveness in human breast cancer by suppressing ERalpha and inducing IL-6 expression. Cancer Res.

[31] Rodriguez BA, Cheng AS, Yan PS, Potter D, Agosto-Perez FJ, Shapiro CL (2008). Epigenetic repression of the estrogen-regulated Homeobox B13 gene in breast cancer. Carcinogenesis.

[32] Tomida S, Yanagisawa K, Koshikawa K, Yatabe Y, Mitsudomi T, Osada H (2007). Identification of a metastasis signature and the DLX4 homeobox protein as a regulator of metastasis by combined transcriptome approach. Oncogene.

[33] Kron K, Liu L, Trudel D, Pethe V, Trachtenberg J, Fleshner N (2012). Correlation of ERG expression and DNA methylation biomarkers with adverse clinicopathologic features of prostate cancer. Clinical cancer research: an official journal of the American Association for Cancer Research.

[34] Huang DW, Sherman BT, Lempicki RA (2009). Systematic and integrative analysis of large gene lists using DAVID bioinformatics resources. Nat Protoc.

[35] Wang Y, He X, Ngeow J, Eng C (2012). GATA2 negatively regulates PTEN by preventing nuclear translocation of androgen receptor and by androgen-independent suppression of PTEN transcription in breast cancer. Hum Mol Genet.

[36] Marsit CJ, Houseman EA, Christensen BC, Gagne L, Wrensch MR, Nelson HH (2010). Identification of methylated genes associated with aggressive bladder cancer. PLoS One.

[37] Gupta RA, Shah N, Wang KC, Kim J, Horlings HM, Wong DJ (2010). Long non-coding RNA HOTAIR reprograms chromatin state to promote cancer metastasis. Nature.

[38] Shah N, Sukumar S (2010). The Hox genes and their roles in oncogenesis. Nat Rev Cancer.

[39] MacKenzie TA, Titus-Ernstoff L, Vacek PM, Geller B, Weiss JE, Goodrich ME (2007). Breast density in relation to risk of ductal carcinoma in situ of the breast in women undergoing screening mammography. Cancer causes & control : CCC.

[40] Dumenil TD, Wockner LF, Bettington M, McKeone DM, Klein K, Bowdler LM (2014). Genome-wide DNA methylation analysis of formalin-fixed paraffin embedded colorectal cancer tissue. Genes Chromosomes Cancer.

[41] Lechner M, Fenton T, West J, Wilson G, Feber A, Henderson S (2013). Identification and functional validation of HPV-mediated hypermethylation in head and neck squamous cell carcinoma. Genome med.

[42] S Moran, M Vizoso, A Martinez-Cardus, A Gomez, X Matias-Guiu, SM Chiavenna et al. Validation of DNA methylation profiling in formalin-fixed paraffin-embedded samples using the Infinium HumanMethylation450 Microarray. Epigenetics: official journal of the DNA Methylation Society. 2014;9(6). 10.4161/epi.28790 PMC4065180 24732293

[43] Dedeurwaerder S, Defrance M, Calonne E, Denis H, Sotiriou C, Fuks F (2011). Evaluation of the Infinium Methylation 450K technology. Epigenomics.

[44] Koestler DC, Li J, Baron JA, Tsongalis GJ, Butterly LF, Goodrich M (2014). Distinct patterns of DNA methylation in conventional adenomas involving the right and left colon. Modern pathology: an official journal of the United States and Canadian Academy of Pathology, Inc.

[45] Roessler J, Ammerpohl O, Gutwein J, Hasemeier B, Anwar SL, Kreipe H (2012). Quantitative cross-validation and content analysis of the 450k DNA methylation array from Illumina. Inc BMC research notes.

[46] Morris TJ, Butcher LM, Feber A, Teschendorff AE, Chakravarthy AR, Wojdacz TK (2014). ChAMP: 450k Chip Analysis Methylation Pipeline. Bioinformatics.

[47] Teschendorff AE, Marabita F, Lechner M, Bartlett T, Tegner J, Gomez-Cabrero D (2013). A beta-mixture quantile normalization method for correcting probe design bias in Illumina Infinium 450 k DNA methylation data. Bioinformatics.

[48] Johnson WE, Li C, Rabinovic A (2007). Adjusting batch effects in microarray expression data using empirical Bayes methods. Biostatistics.

[49] Wilkerson MD, Hayes DN (2010). ConsensusClusterPlus: a class discovery tool with confidence assessments and item tracking. Bioinformatics.

[50] Jones JL (2006). Overdiagnosis and overtreatment of breast cancer: progression of ductal carcinoma in situ: the pathological perspective. Breast cancer research : BCR.

[51] Huang DW, Sherman BT, Lempicki RA (2009). Bioinformatics enrichment tools: paths toward the comprehensive functional analysis of large gene lists. Nucleic Acids Res.

[52] Squazzo SL, O’Geen H, Komashko VM, Krig SR, Jin VX, Jang SW (2006). Suz12 binds to silenced regions of the genome in a cell-type-specific manner. Genome Res.

[53] Schlesinger Y, Straussman R, Keshet I, Farkash S, Hecht M, Zimmerman J (2007). Polycomb-mediated methylation on Lys27 of histone H3 pre-marks genes for de novo methylation in cancer. Nat Genet.

[54] Lee TI, Jenner RG, Boyer LA, Guenther MG, Levine SS, Kumar RM (2006). Control of developmental regulator’s by polycomb in human embryonic stem cells. Cell.

[55] Bracken AP, Dietrich N, Pasini D, Hansen KH, Helin K (2006). Genome-wide mapping of Polycomb target genes unravels their roles in cell fate transitions. Gene Dev.

[56] Karolchik D, Hinrichs AS, Furey TS, Roskin KM, Sugnet CW, Haussler D (2004). The UCSC Table Browser data retrieval tool. Nucleic Acids Res.

