# Supplementary material for: DNA methylation in ductal carcinoma in situ related with future development of invasive breast cancer
Source: Clin Epigenetics. 2015 Jul 25;7(1):75. doi: 10.1186/s13148-015-0094-0 (PMC4514996; doi:10.1186/s13148-015-0094-0)
Supplement: Additional file 1: — Supplemental Figure S1. Diagram of analytic strategy used to identify deregulated epigenetic patterns in DCIS and their relation with invasive breast cancer development. [file 13148_2015_94_MOESM1_ESM.pptx]

## Slide 1
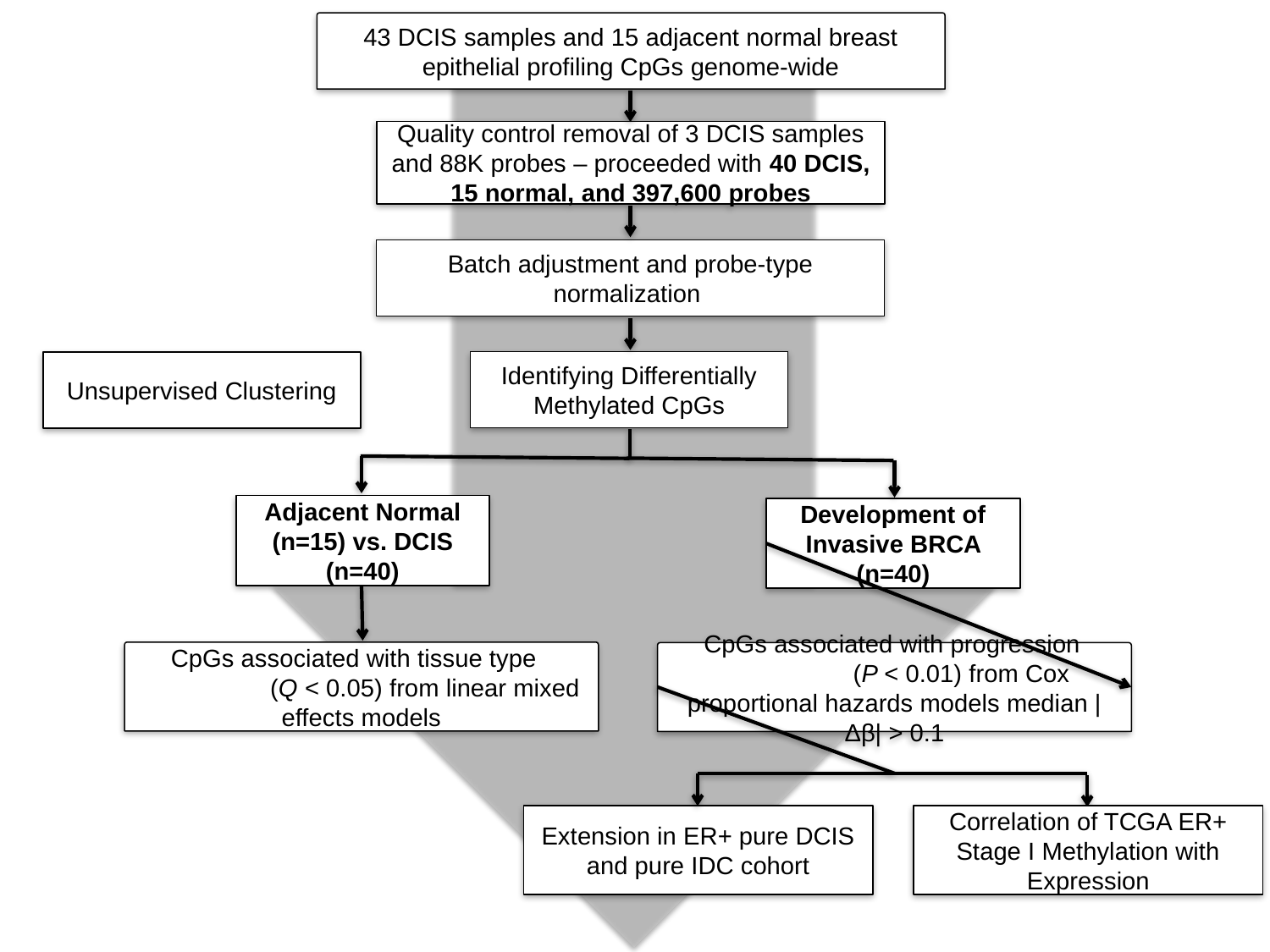

43 DCIS samples and 15 adjacent normal breast epithelial profiling CpGs genome-wide
Quality control removal of 3 DCIS samples and 88K probes – proceeded with 40 DCIS, 15 normal, and 397,600 probes
Batch adjustment and probe-type normalization
Identifying Differentially Methylated CpGs
Unsupervised Clustering
Adjacent Normal (n=15) vs. DCIS (n=40)
Development of Invasive BRCA (n=40)
CpGs associated with tissue type 		(Q < 0.05) from linear mixed effects models
CpGs associated with progression		 (P < 0.01) from Cox proportional hazards models median |Δβ| > 0.1
Extension in ER+ pure DCIS and pure IDC cohort
Correlation of TCGA ER+ Stage I Methylation with Expression
