# Supplementary figures and images for: DNA methylation in ductal carcinoma in situ related with future development of invasive breast cancer
Source: Clin Epigenetics. 2015 Jul 25;7(1):75. doi: 10.1186/s13148-015-0094-0 (PMC4514996; doi:10.1186/s13148-015-0094-0)

## Slide 1
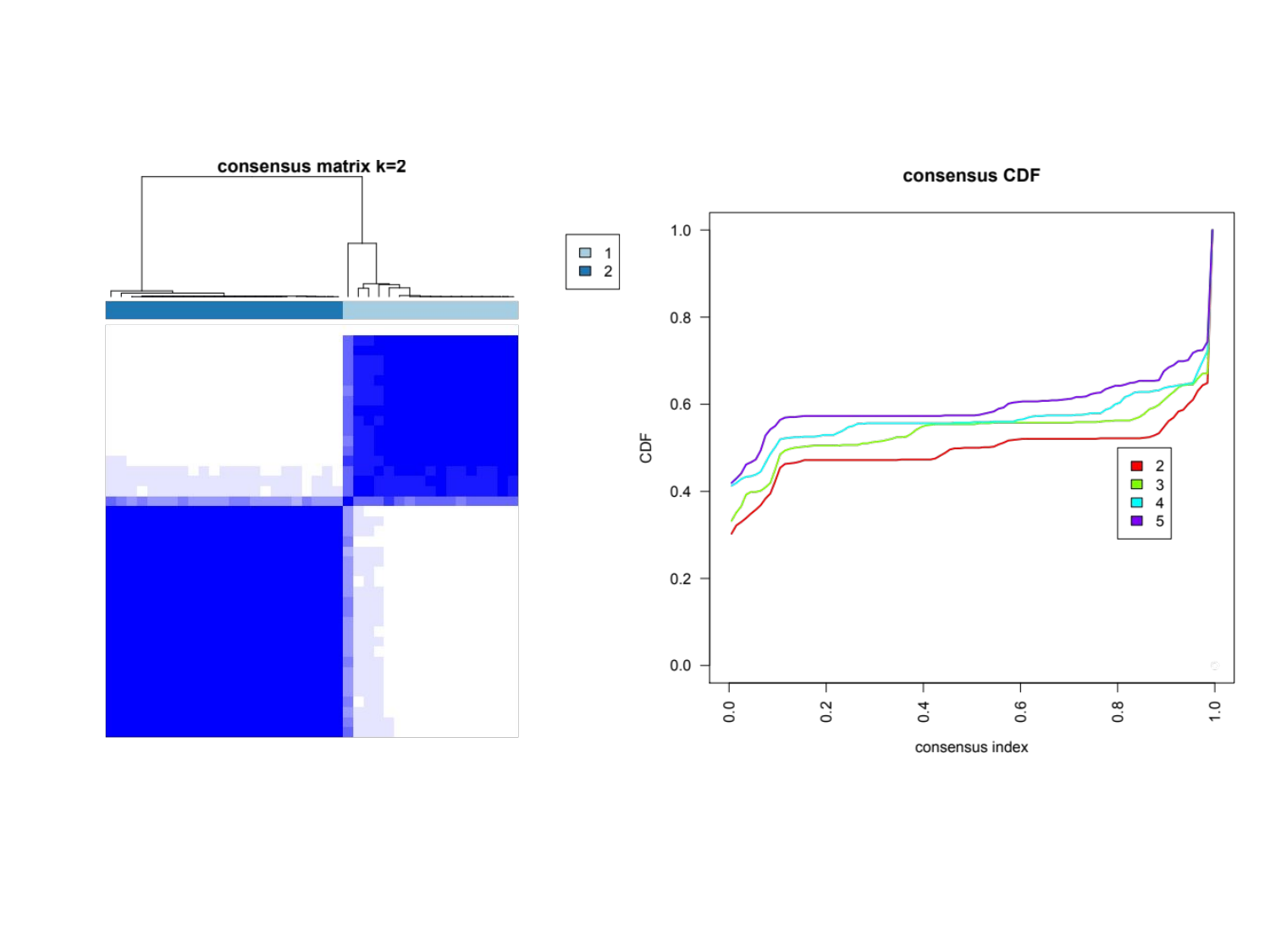

Supplement: Additional file 2: — Supplemental Figure S2. Consensus clustering of 40 samples and 10,000 most variable CpGs identified 2 clusters as the optimal number. [file 13148_2015_94_MOESM2_ESM.pptx]

## Slide 1
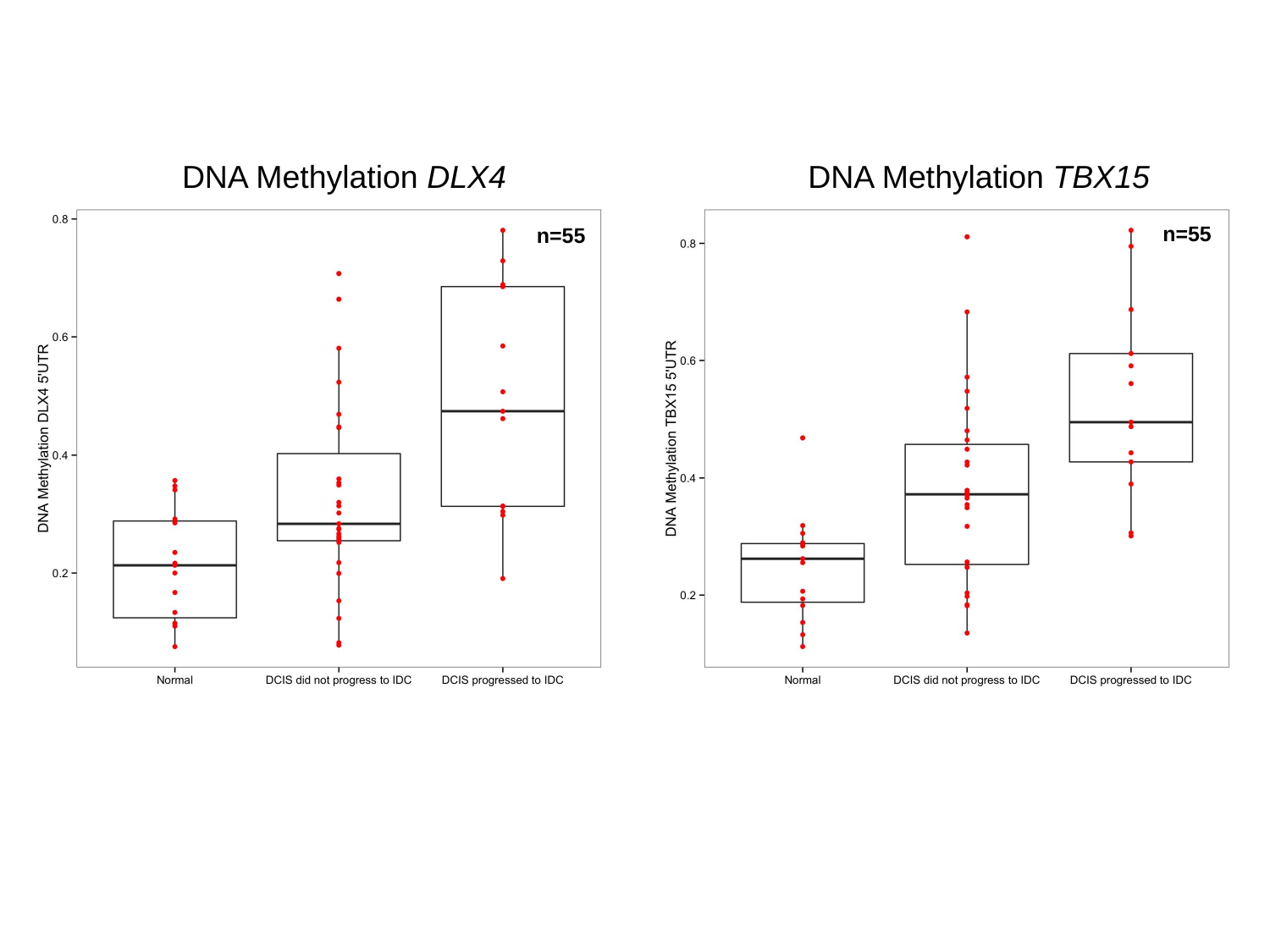

DNA Methylation DLX4
DNA Methylation TBX15
n=55
n=55

Supplement: Additional file 5: — Supplemental Figure S4. A total of 276 CpGs among the 641 progression-related loci experienced additional DNA methylation deregulation in the same direction that differentiated DCIS from normal-adjacent tissue. [file 13148_2015_94_MOESM5_ESM.pptx]
