# Supplementary material for: DNA methylation in ductal carcinoma in situ related with future development of invasive breast cancer
Source: Clin Epigenetics. 2015 Jul 25;7(1):75. doi: 10.1186/s13148-015-0094-0 (PMC4514996; doi:10.1186/s13148-015-0094-0)
Supplement: Additional file 3: — Supplemental Figure S3. Results from the locus-by-locus examination of differential methylation between DCIS and normal-adjacent to DCIS tissues. [file 13148_2015_94_MOESM3_ESM.pptx]

## Slide 1
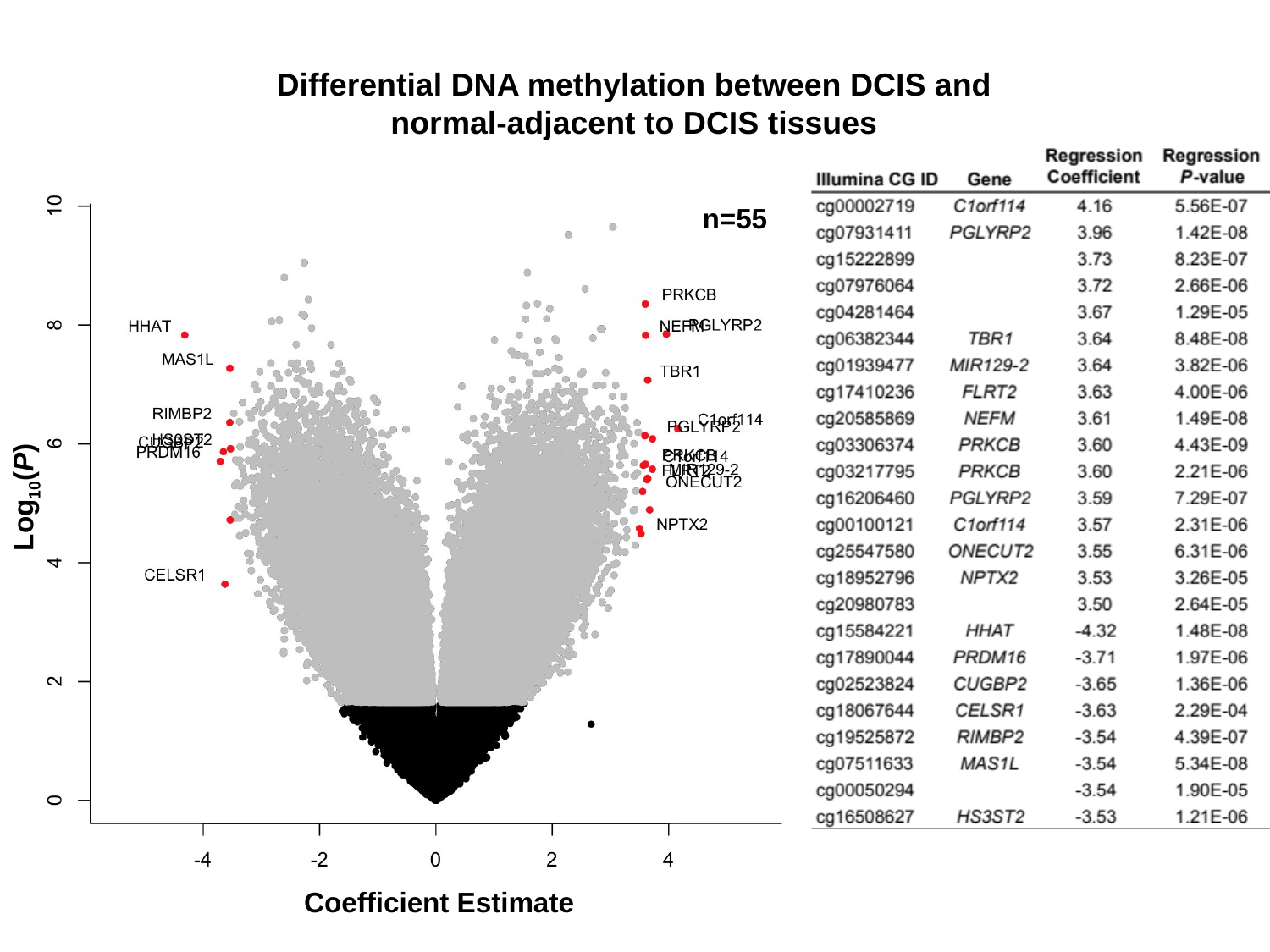

Differential DNA methylation between DCIS and normal-adjacent to DCIS tissues
n=55
Log10(P)
Coefficient Estimate
