# Supplementary material for: DNA methylation in ductal carcinoma in situ related with future development of invasive breast cancer
Source: Clin Epigenetics. 2015 Jul 25;7(1):75. doi: 10.1186/s13148-015-0094-0 (PMC4514996; doi:10.1186/s13148-015-0094-0)
Supplement: Additional file 11: — Supplemental Figure S5. Unsupervised clustering heat map based on Manhattan distance and average linkage of CpGs that track to the 72 genes and 28 genes. [file 13148_2015_94_MOESM11_ESM.pptx]

## Slide 1
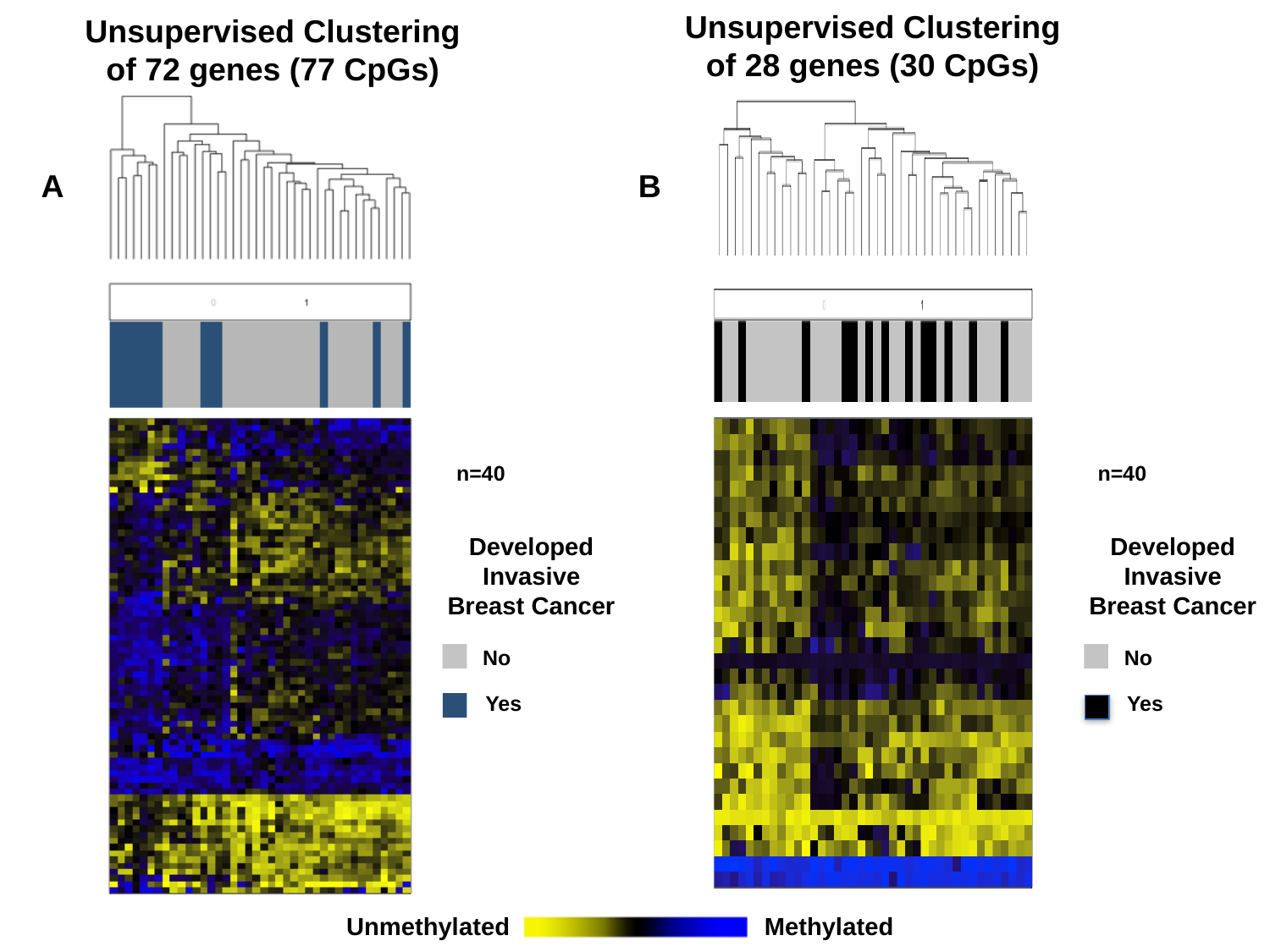

Unsupervised Clustering of 28 genes (30 CpGs)
Unsupervised Clustering of 72 genes (77 CpGs)
A
B
n=40
n=40
Developed Invasive Breast Cancer
Developed Invasive Breast Cancer
No
No
Yes
Yes
Unmethylated
Methylated
